# Vitreoretinal interface abnormalities in fellow eyes of patients with a macular hole

**DOI:** 10.20407/fmj.2023-016

**Published:** 2024-02-15

**Authors:** Koji Ueoka, Tadashi Mizuguchi, Masayuki Horiguchi, Yasuki Ito

**Affiliations:** 1 Department of Ophthalmology, Toyokawa City Hospital, Toyokawa, Aichi, Japan; 2 Department of Ophthalmology, Fujita Health University, School of Medicine, Toyoake, Aichi, Japan

**Keywords:** Fellow eye, Macular hole, Optical coherence tomography, Vitreoretinal interface abnormalities

## Abstract

**Objectives:**

This study aimed to determine the incidence of bilateral macular holes (MHs), and to investigate the incidence and characteristics of retinal abnormalities in the fellow eyes, using high-definition optical coherence tomography (OCT).

**Methods:**

Medical records from 724 consecutive patients diagnosed with full-thickness MHs, who underwent surgery at Fujita Health University Hospital from May 2015 to March 2022, were retrospectively reviewed. Based on the exclusion criteria, 658 patients with idiopathic MHs were included. B-scan OCT images of the fellow eyes were used to identify vitreoretinal interface abnormalities, such as MHs, epiretinal membranes (ERMs), vitreomacular traction syndrome (VMTS) and lamellar macular holes (LMHs).

**Results:**

The mean age of the patients was 65.9±9.0 years. There were 292 males (44.4%) and 366 females (55.6%). There were 39 fellow eyes with MHs (5.9%), 77 with ERMs (11.7%), 32 with VMTS (4.9%) and 11 with LMHs (1.7%). Thirty-four fellow eyes (5.2%) could not be classified and 469 fellow eyes were normal (71.3%). Furthermore, the 39 bilateral MHs were divided into four stages according to the Gass classification, as follows: stage 1, 32 eyes (82.1%); stage 2, none (0%); stage 3, 5 eyes (12.8%); and stage 4, 2 eyes (5.1%).

**Conclusions:**

Vitreoretinal interface abnormalities, such as MHs, ERMs, VMTS and LMHs, are more common in the fellow eyes of patients with MHs than in the healthy population. The fellow eyes of patients with MHs should be carefully followed using OCT.

## Introduction

Macular holes (MHs), epiretinal membranes (ERMs) and vitreomacular traction syndrome (VMTS), which are vitreomacular interface abnormalities, are conditions in which the foveal retina is deformed by vitreous traction that causes distortion, hyperopia and decreased visual acuity. A recent report using optical coherence tomography (OCT) revealed that tangential vitreoretinal traction is mainly caused by the development of a full-thickness MH.^1^ Surgery can minimize distortion, improve macroscopic vision (in other words, reduce the likelihood of the left and right eyes seeing objects as different sizes) and improve visual acuity in these conditions. In previous studies, OCT revealed that vitreomacular interface abnormalities, such as ERMs and VMTS in fellow eyes, increased the risk of developing a full-thickness MH.^2–5^ Lamellar macular holes (LMHs) are characterized by irregular foveal contours, damage to the inner layer of the foveal retina and separation of the outer and inner layers of the retina.^6^ Depending on the pathology, LMHs can be divided into those caused by vitreous traction on the retina and those caused by degeneration; however, it has recently been reported that the effects of vitreoretinal traction cannot be ignored in either type of LMH.^7^ The effects of surgery on LMHs have also been reported.^8,9^ To guide postoperative follow-up, it is important to determine whether these vitreomacular interface abnormalities are more common in the fellow eyes of patients with an MH than in normal individuals.

To date, the incidence of bilateral MHs and the risk of an MH in the fellow eye have been reported to vary from 7.0% to 16.7%.^2,4,10–14^ Posterior vitreous detachments (PVDs), especially parafoveal PVDs (VMTS) and ERMs, have been reported as risk factors for MHs in fellow eyes. However, few studies have been conducted with a large number of patients and few have examined a large number of cases of MH fellow eyes using swept-source OCT (SS-OCT), which can capture higher-resolution images than the previous generation of OCT (i.e., spectrum-domain OCT).

To the best of our knowledge, there is little in the literature regarding the incidence of LMHs in the fellow eyes of patients with an MH. In particular, there are few reports on the incidence of LMHs in MH fellow eyes as determined using high-definition OCT. In this study, we used SS-OCT to examine the incidence of vitreomacular interface abnormalities, including LMHs, in the fellow eyes of patients with an MH operated upon at our facility. This study aimed to determine the incidence of bilateral MH, and to investigate the incidence and OCT-based characteristics of retinal abnormalities in the fellow eyes.

## Methods

### Study design and participants

This retrospective, observational study used data collected from medical records. This study was approved by the Ethics Committee of Fujita Health University Hospital (approval number: HM16-089) and conformed to the requirements of the US Health Insurance Portability and Accountability Act of 1996. This study was conducted according to the principles of the Declaration of Helsinki and the STROBE guidelines. Written informed consent was obtained from all the patients.

The medical records of 724 consecutive patients (757 eyes) who underwent MH surgery from May 2015 to March 2022 were retrospectively reviewed. There were 39 patients with bilateral MHs. In those cases, the first eye operated upon was specified as the affected (MH) eye, and the second operated upon was defined as the fellow eye.

### Inclusion and exclusion criteria

The inclusion criterion was an initial surgery for idiopathic MH.

**Figure 1:**
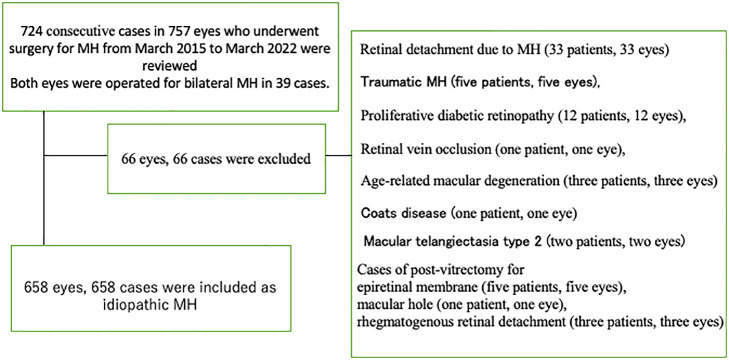
Inclusion and exclusion criteria. From 724 consecutive patients (724 eyes) who underwent surgery for a macular hole (MH), 66 eyes of 66 patients were excluded based on the exclusion criteria. Therefore, in total, 658 eyes of 658 patients were included as having an idiopathic MH.

The exclusion criteria, and numbers of affected patients, were as follows (in each case, 1 eye per patient was affected): retinal detachment due to MH (33 patients); traumatic MH (5 patients); uveitis, or other intraocular inflammation (none); and other retinal disorders that might affect visual acuity or macular morphology. The latter disorders included: proliferative diabetic retinopathy (12 patients); retinal vein occlusion (1 patient); age-related macular degeneration (3 patients), Coats disease (1 patient) and macular telangiectasia type 2 (2 patients). Patients who underwent post-vitrectomy treatment for an ERM (5 patients, 5 eyes), MH (1 patient, 1 eye) or rhegmatogenous retinal detachment (3 patients, 3 eyes) were also excluded. After excluding those cases, 658 patients were included (Figure 1).

### OCT examination methods

B-scan images of the macular region (7×7 mm) of both eyes were obtained by a single technician using SS-OCT (Topcon DRI OCT Triton^®^; Topcon Corp., Tokyo, Japan) when the patient was examined for the MH in the first eye.

### Definition of the disease

**Figure 2:**
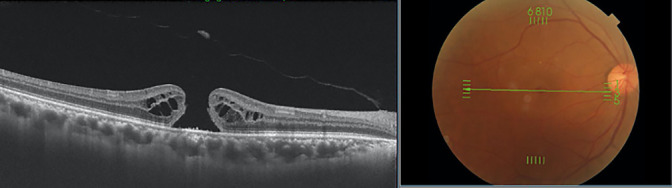
Representative case of the fellow eye of a patient with a macular hole. A B-scan tomographic image is presented. Posterior vitreous detachment had occurred in the macula, and the operculum is apparent anteriorly in the retina. A full-thickness macular hole with a fluid cuff was observed; this was considered a stage-3 macular hole.

**Figure 3:**
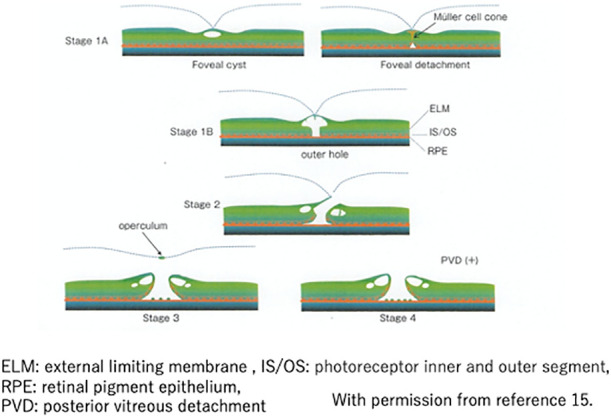
Classification of macular holes with optical coherence tomography. In stage 1A, when the fovea experiences traction due to perifoveal posterior vitreous detachment (PVD), a cyst forms between the superficial glia and the outer nuclear layer (foveal cyst). Another observed pattern is micro-retinal detachment at the photoreceptor inner and outer segment (IS/OS) level (foveal detachment). The apex of the foveal detachment may penetrate into the outer nuclear layer, resembling a fissure. In stage 1B, the cyst enlarges and cracks form in the outer nuclear layer. As these cracks expand, they transform into an outer hole. In stage 2, the roof of the cyst is pulled into a valve-like shape, creating a noticeable gap. In stage 3, the vitreal cortex detaches at the fovea, with the operculum becoming attached to it. Finally, in stage 4, complete PVD occurs.

MHs were diagnosed in accordance with the International Vitreomacular Traction Study Group classification of vitreomacular traction (Figure 2). MH-staging, according to the Gass classification, was performed based on the presence or absence of parafoveal PVD on OCT (Figure 3).^15^ Stage 4 MH and PVD were determined by examining surgical reports and medical chart fundus findings. Furthermore, stage-1 MHs were divided into two types, according to the macular morphology on OCT.

### Stage 1A

An inner retinal cyst was defined as a separation between the inner nuclear and outer plexiform layers, with attachment of the hyaloid to the retina. Foveal detachment was defined as the elevation of the external limiting membrane and ellipsoid zone, with attachment of the hyaloid to the retina (Figure 3).

### Stage 1B

**Figure 4:**
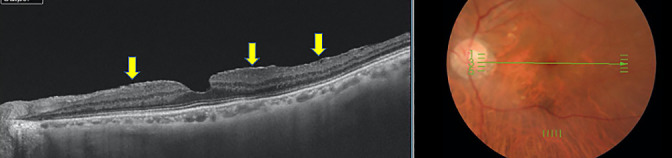
Representative case of an epiretinal membrane in the fellow eye of a patient with an idiopathic macular hole. An epiretinal membrane and folds, which are hyperreflective on the retina, are visible (yellow arrows).

**Figure 5:**
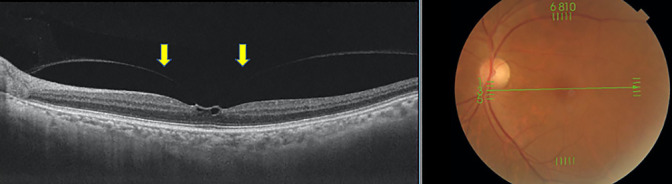
Representative case of vitreomacular traction syndrome in the fellow eye of a patient with an idiopathic macular hole. Traction and foveal distortion of the retina, due to the vitreal cortex associated with the perifoveal posterior vitreous detachment, can be seen (yellow arrows).

An outer hole was defined as a connection between the inner retinal cyst and outer retinal detachment, with a wider defect of the outer retina and intact retinal surface (Figure 3). An ERM was defined based on the presence of hyper-refractive tissue above the retina on OCT images (Figure 4). VMT was defined as an anatomic distortion of the fovea in combination with perifoveal PVD, as described by Duker et al.^16^ (Figure 5).

**Figure 6:**
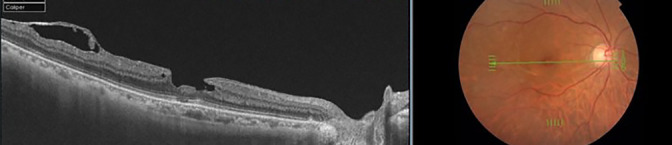
Representative case of a lamellar macular hole in the fellow eye of a patient with an idiopathic macular hole. An irregular foveal outline, burrowing of the inner foveal retina and separation of the outer and inner retina are visible. Unlike a full-thickness macular hole, there is no ellipsoid-zone defect and no full-thickness foveal defect.

Continuing with Duker et al.’s terminology, LMHs were characterized by irregular foveal contours, damage to the inner layer of the foveal retina and separation of the outer and inner layers of the retina.^16^ Unlike full-thickness MHs, LMHs were defined by the absence of ellipsoid-zone defects or full-thickness foveal defects (Figure 6). Furthermore, MH at stage 2 or higher was considered a full-thickness MH. Changes in the inner layer of the retina due to vitreoretinal traction, such as an inner retinal cyst and inner foveal split, were classified as VMTS; changes in the outer layer of the retina, such as serous retinal detachment, were also counted as VMTS.

When we classified retinal abnormalities, duplication was not allowed. For example, if MH, ERM, LMH and VMTS existed at the same time, the patient would be classified as having MH. MH was classified first, LMH second, VMTS third and ERM fourth. However, MH stage 1 and VMTS were treated as the same disease, since VMTS and stage-1 MH had the same definition.^16^ VMTS was also included when counting the number of MHs in this study.

### Assessment of OCT findings

The SS-OCT images of fellow eyes were interpreted and classified by two vitreoretinal specialists (M.H. and Y.I.) who were blinded to patient information.

## Results

The mean age of the patients was 65.9±9.0 years. There were 292 males (44.4%) and 366 females (55.6%). Thirty-nine patients had bilateral MHs at their initial visit (5.9%). Among those 39 fellow eyes, the MHs in 32 eyes (82.1%) were classified as stage 1; in 5 eyes (12.8%) as stage 3; and in 2 eyes (5.1%) as stage 4. The stage-1 MHs were divided into stage 1A and stage 1B. Stage 1A comprised an inner retinal cyst in 23 eyes and an outer retinal defect in 7 eyes. Two cases with no inner retinal abnormality and only an outer retinal hole were classified as stage 1B. The other vitreoretinal interface abnormalities that were observed were as follows: 77 eyes had an ERM (11.7%), 32 eyes had VMTS (4.9%) and 11 eyes had an LMH (1.7%). Thirty-four eyes (5.2%) could not be classified based on the OCT images and 469 eyes (71.3%) were normal.

## Discussion

In this study, we investigated whether vitreomacular interface abnormalities, especially MHs, ERMs, VMTS and LMHs, were more common in the fellow eyes of patients with MH than in the normal population. This study revealed that the rate of bilateral MHs (5.5%) was greater than the rate of unilateral MHs (0.008%) in the general population.^6^ The percentage of cases of development of a full-thickness MH in the fellow eye of patients with a unilateral MH varied from 2.8% to 15.6% in previous reports.^2,5,13,14^ Previous studies with longer observation periods reported higher rates of MH development in the fellow eyes. In this study, the fellow eye was examined using OCT at the initial visit, when the first eye was examined for MH. Studies that reported the rates of partial-thickness holes (i.e., macular cysts and foveal split) in their analysis overestimated the rates compared with studies that excluded partial-thickness holes.

The incidence of MH in the fellow eye reported by Ezra et al. was the highest at 15%.^13^ When PVD occurs, traction on the macula causes a MH, so the fellow eye of participants who had not have PVD yet had a higher probability of developing MH, compared with other reports including fellow eyes with PVD. Thus, the incidence was higher in their report than in other reports.

Regarding the influence of race on the incidence of MHs, Ali et al. reported that Asian-Americans had a 1.77-fold higher incidence than did Caucasians.^17^ The higher rate of vitreoretinal interface abnormalities in MH fellow eyes in our study is consistent with the reports of Kumagai et al.^12^ and Chahblani et al.^5^ in the Asian population.

The population-based incidence of ERMs has been reported to be 5.7%,^18^ and the incidence of an ERM in MH fellow eyes ranges from 25.4% to 28.1%.^5,18^ The incidence in the present study (11.7%) is lower than in the previous reports.^5,18^ The previous studies permitted duplication of ERM and another vitreoretinal interface abnormality, but the present study did not permit such duplication, so the incidence may have been underestimated.

The population-based incidence of VMTS is 2.3%^19^ and the reported incidence of VMTS in MH fellow eyes ranges from 4.9% to 17%.^3,5,12,18^ In the present study, the incidence was 4.9%, which is higher than the population-based one. We did not count eyes with vitreoretinal attachments with parafoveal PVD, and only patients with anatomical changes in the fovea were counted as having VMTS. Therefore, the incidence of VMTS may have been underestimated in our study.

To the best of our knowledge, a population-based incidence of LMH has not been reported previously. In the present study, the incidence of LMH in the fellow eye was 1.7%.

In this study, we did not count duplications of ERMs, MHs or LMHs in the classification of fellow eyes, so it is possible that the incidence of each retinal abnormality was underestimated compared with that in past reports with duplications.

Why is there a high incidence of vitreoretinal interface abnormalities in fellow eyes with MH? It has been reported that OCT abnormalities of the vitreoretinal interfaces in fellow eyes with MH are structural changes that increase the risk for MH formation.^2–5^ Perifoveal PVD has a significant effect on VMTS and full-thickness MH formation. Takahashi et al. reported that fellow eyes with foveolar detachment and perifoveal PVD may be at high risk, and fellow eyes with foveolar detachment and inner foveal splits might be at an even higher risk for progression to MH formation.^3^ Recently, Bringmann et al.^20^ described different modes of MH formation and emphasized that MH formation is caused by the disruption of both Müller cell cones and the external limiting membrane. Perifoveal PVD and foveal abnormalities, such as inner foveal split and foveal serous retinal detachment, which are the result of previous traction, promote the formation of full-thickness MHs induced by subsequent ERM-induced tangential traction on the retinal surface. Some LMHs may develop into full-thickness MHs, as a result of tangential traction.^21–24^

This was a retrospective, single-facility study with a large number of cases (658 eyes) investigated using SS-OCT, which revealed the incidence of LMHs in fellow eyes of patients with an MH. The main limitation of the study is that it investigated the incidence of vitreoretinal interface abnormalities in the fellow eyes of affected patients only at the initial visit, without follow-up. Therefore, we could not determine the onset risk of developing full-thickness MH with the passage of time. Long-term follow-up and serial OCT examinations are required to determine the degree of PVD and VMTS, the thickness and extent of ERMs and the morphology of LMHs affecting the subsequent formation of full-thickness MHs.

Our study revealed a high rate (19.3%) of vitreoretinal interface abnormalities in the fellow eyes of patients with an MH. Therefore, the fellow eyes of such patients should be carefully followed using high-resolution OCT.

## Previous Presentation

This manuscript was presented in part at the 61^st^ annual meeting of the Japanese Retina and Vitreous Society held on December 2–4, 2022 in Osaka, Japan.

## Availability of Data and Materials

The datasets generated and analyzed during the current study are not publicly available due to our plan to submit another study based on the same data, but the data are available from the corresponding author on reasonable request.

## References

[1] Steel DH, Lotery AJ. Idiopathic vitreomacular traction and macular hole: a comprehensive review of pathophysiology, diagnosis, and treatment. Eye (Lond) 2013; 27: S1–S21. 24108069 10.1038/eye.2013.212 PMC3797995

[2] Choi JH, Kim KT, Kang SW, Bae K, Lee SE, Kim AY. Development of idiopathic macular hole in fellow eyes: spectral domain optical coherence tomography features. Retina 2020; 40: 765–772. 30664122 10.1097/IAE.0000000000002439

[3] Takahashi A, Yoshida A, Nagaoka T, Kagokawa H, Kato Y, Takamiya A, Sato E, Yokota H, Ishiko S, Hirokawa H. Macular hole formation in fellow eyes with a perifoveal posterior vitreous detachment of patients with a unilateral macular hole. Am J Ophthalmol 2011; 151: 981–989.e4. 21514922 10.1016/j.ajo.2011.01.011

[4] Michalewska Z, Michalewski J, Sikorski BL, Kałuzny JJ, Wojtkowski M, Adelman RA, Nawrocki J. A study of macular hole formation by serial spectral optical coherence tomography. Clin Exp Ophthalmol 2009; 37: 373–383. 19594564 10.1111/j.1442-9071.2009.02041.x

[5] Chhablani J, Kumar K, Ali TR, Narayanan R. Spectral-domain optical coherence tomography features in fellow eyes of patients with idiopathic macular hole. Eur J Ophthalmol 2014; 24: 382–386. 24242224 10.5301/ejo.5000386

[6] Hubschman JP, Govetto A, Spaide RF, Schumann R, Steel D, Figueroa MS, Sebag J, Gaudric A, Staurenghi G, Haritoglou C, Kadonosono K, Thompson JT, Chang S, Bottoni F, Tadayoni R. Optical coherence tomography-based consensus definition for lamellar macular hole. Br J Ophthalmol 2020; 104: 1741–1747. 32107208 10.1136/bjophthalmol-2019-315432

[7] Govetto A, Dacquay Y, Farajzadeh M, Platner E, Hirabayashi K, Hosseini H, Schwartz SD, Hubschman JP. Lamellar macular hole: two distinct clinical entities? Am J Ophthalmol 2016; 164: 99–109. 26898164 10.1016/j.ajo.2016.02.008

[8] Michalewska Z, Michalewski J, Odrobina D, Pikulski Z, Cisiecki S, Dziegielewski K, Nawrocki J. Surgical treatment of lamellar macular holes. Graefes Arch Clin Exp Ophthalmol 2010; 248: 1395–1400. 20461401 10.1007/s00417-010-1400-3

[9] Shiraga F, Takasu I, Fukuda K, Fujita T, Yamashita A, Hirooka K, Shirakata Y, Morizane Y, Fujiwara A. Modified vitreous surgery for symptomatic lamellar macular hole with epiretinal membrane containing macular pigment. Retina 2013; 33: 1263–1269. 23594723 10.1097/IAE.0b013e31828bcb61

[10] Forsaa VA, Lindtjørn B, Kvaløy JT, Frøystein T, Krohn J. Epidemiology and morphology of full-thickness macular holes. Acta Ophthalmol 2018; 96: 397–404. 29197164 10.1111/aos.13618

[11] McCannel CA, Ensminger JL, Diehl NN, Hodge DN. Population-based incidence of macular holes. Ophthalmology 2009; 116: 1366–1369. 19576500 10.1016/j.ophtha.2009.01.052 PMC2867090

[12] Kumagai K, Ogino N, Hangai M, Larson E. Percentage of fellow eyes that develop full-thickness macular hole in patients with unilateral macular hole. Arch Ophthalmol 2012; 130: 393–394. 22411672 10.1001/archopthalmol.2011.1427

[13] Ezra E, Wells JA, Gray RH, Kinsella FM, Orr GM, Grego J, Arden GB, Gregor ZJ. Incidence of idiopathic full-thickness macular holes in fellow eyes. A 5-year prospective natural history study. Ophthalmology 1998;105: 353–359. 10.1016/s0161-6420(98)93562-x 9479299

[14] Niwa H, Terasaki H, Ito Y, Miyake Y. Macular hole development in fellow eyes of patients with unilateral macular hole. Am J Ophthalmol 2005; 140: 370–375. 16084790 10.1016/j.ajo.2005.03.070

[15] Kishi S. Optical coherence tomography in diagnosis of retinal disease. 3rd ed. Tokyo: Elsevier Japan; 2014: 65.

[16] Duker JS, Kaiser PK, Binder S, de Smet MD, Gaudric A, Reichel E, Sadda SR, Sebag J, Spaide RF, Stalmans P. The international vitreomacular traction study group classification of vitreomacular adhesion, and macular hole. Ophthalmology 2013; 120: 2611–2619. 24053995 10.1016/j.ophtha.2013.07.042

[17] Ali FS, Stein JD, Blachley TS, Ackley S, Stewart JM. Incidence of and risk factors for developing idiopathic macular hole among a diverse group of patients throughout the United States. JAMA Opthalomol 2017; 135: 299–305. 10.1001/jamaophthalmol.2016.5870 PMC5470400 28208188

[18] Lindtjørn B, Krohn J, Forsaa VA. Optical coherence tomography features and risk of macular hole formation in the fellow eye. BMC Ophthalmol 2021; 29; 21: 351. 10.1186/s12886-021-02111-1 PMC8482611 34587925

[19] Shao L, Zhang C, Dong L, Zhou WD, Zhang RH, Wei WB. Prevalence and associations of vitreomacular traction: The Beijing Eye Study. Int J Gen Med 2021; 14: 7059–7064. 34707397 10.2147/IJGM.S333010 PMC8544120

[20] Bringmann A, Unterlauft JD, Barth T, Wiedemann R, Rehak M, Wiedemann P. Different modes of full-thickness macular hole formation. Exp Eye Res 2021; 202: 108393. 33301774 10.1016/j.exer.2020.108393

[21] Bottoni F, Deiro AP, Giani A, Orini C, Cigada M, Staurenghi G. The natural history of lamellar macular holes: a spectral domain optical coherence tomography study. Graefes Arch Clin Exp Ophthalmol 2013; 251: 467–475. 22569859 10.1007/s00417-012-2044-2

[22] Asaad SZ. Lamellar macular holes: evolving concepts and propensity for progression to full thickness macular hole. Int J Retina Vitreous 2020; 6: 45. 33005441 10.1186/s40942-020-00252-x PMC7526127

[23] Tsai CY, Hsieh YT, Yang CM. Epiretinal membrane-induced full-thickness macular holes: the clinical features and surgical outcomes. Retina 2016; 36: 1679–1687. 26934453 10.1097/IAE.0000000000000999

[24] Pang CE, Spaide RF, Freund KB. Epiretinal proliferation seen in association with lamellar macular holes: a distinct clinical entity. Retina 2014; 34: 1513–1523. 24732699 10.1097/IAE.0000000000000163

